# Domestic Summary

**Published:** 1839-01-19

**Authors:** 


### DOMESTIC SUMMARY.

*Singular case of extra-uterine Pregnancy.*—The following curious and interesting case of extra-uterine pregnancy, is reported to us in a letter of 10th December, 1838, from Wm. F. Baldwin, M. D., of Union Springs, Macon county, Alabama, near which place it occurred. Dr. Baldwin was unable to obtain any history of the case previous to death.

"Patient, a negro woman, aged about twenty-five years. Post-mortem examination disclosed the following facts. Commenced the incision about two inches above the umbilicus; in cutting through the abdominal parietes, I first came in contact with the placenta, adhering to the parietes of the abdomen for about two inches around the umbilicus. On opening the abdomen I found the child in the following position: it was lying parallel with the abdomen of its mother, with its head occupying the epigastric region, being in contact with the stomach—its right side opposite the umbilicus, and the left to the intestines. It was as large a child as I have ever seen at birth, and perfectly formed, except the head, which was enormously distended with gas—all the bones of the head were broken into several pieces, which must have been occasioned by the inordinate contractions of the abdominal muscles, at the period at which labour should have taken place. The umbilical cord was of the usual size and length, inserted into the placenta opposite the umbilicus.

"The uterus was about the size of a goose-egg: its cavity containing a small quantity of thin glairy matter. Saw no particular derangement of the abdominal viscera. The child was supposed to have been dead some ten or twelve days.

"The above examination was witnessed by several respectable gentlemen."—*Southern Med. and Surg. Jour., for January.*

